# Lack of ADAM2, CALR3 and SAGE1 Cancer/Testis Antigen Expression in Lung and Breast Cancer

**DOI:** 10.1371/journal.pone.0134967

**Published:** 2015-08-07

**Authors:** Emeaga Maheswaran, Christina B. Pedersen, Henrik J. Ditzel, Morten F. Gjerstorff

**Affiliations:** 1 Department of Cancer and Inflammation Research, Institute for Molecular Medicine (IMM), University of Southern Denmark, Odense, Denmark; 2 Department of Oncology, Odense University Hospital, Odense, Denmark; University of Navarra, SPAIN

## Abstract

Immunotherapy is emerging as a supplement to conventional cancer treatment, and identifying antigen targets for specific types of cancer is critical to optimizing therapeutic efficacy. Cancer/testis antigens are highly promising targets for immunotherapy due to their cancer-specific expression and antigenic properties, but the expression patterns of most of the more than 200 identified cancer/testis antigens in various cancers remain largely uncharacterized. In this study, we investigated the expression of the cancer/testis antigens ADAM2, CALR3 and SAGE1 in lung and breast cancer, the two most frequent human cancers, with the purpose of providing novel therapeutic targets for these diseases. We used a set of previously uncharacterized antibodies against the cancer/testis antigens ADAM2, CALR3 and SAGE1 to investigate their expression in a large panel of normal tissues as well as breast and lung cancers. Staining for the well-characterized MAGE-A proteins was included for comparison. Immunohistochemical staining confirmed previous mRNA analysis demonstrating that ADAM2, CALR3 and SAGE1 proteins are confined to testis in normal individuals. Negative tissues included plancenta, which express many other CT antigens, such as MAGE-A proteins. Surprisingly, we detected no ADAM2, CALR3 and SAGE1 in the 67 lung cancers (mainly non-small lung cancer) and 189 breast cancers, while MAGE-A proteins were present in 15% and 7–16% of these tumor types, respectively. Treatment with DNA methyltransferase inhibitors has been proposed as an attractive strategy to increase the expression of cancer/testis antigens in tumors before immunotargeting; however, neither ADAM2, CALR3 nor SAGE1 could be significantly induced in lung and breast cancer cell lines using this strategy. Our results suggest that ADAM2, CALR3 and SAGE1 cancer/testis antigens are not promising targets for immunotherapy of breast and lung cancer.

## Introduction

Modulation of the immune system in cancer patients has shown to efficiently generate anti-tumor immune responses, but selection of targets for effective and specific intervention remains challenging. The unique expression pattern and immunogenic properties of cancer/testis (CT) antigens make them ideal targets for different types of cancer immunotherapy, such as vaccination and adoptive transfer with recombinant T-cell receptor-transduced T cells. CT antigens are male germ cell proteins ectopically expressed in various malignancies [1–3]. Male germ cells are devoid of HLA-class I molecules and cannot present antigens to T cells. Therefore, CT antigens can be considered tumor-specific neo-antigens when expressed in tumor cells, and have the capacity to elicit immune responses that are strictly tumor-specific. This is consistent with the frequent observations of cellular and humoral immune responses to CT antigens in cancer patients [4–8]. Thus, cancer/testis antigens suggest the promise of highly specific immunotargeting of human cancers.

More than 200 different CT antigens have been identified (CTDatabase, http://www.cta.lncc.br), but only a small number of these have been investigated for expression profiles. Although some CT antigens tend to be co-expressed in a subset of tumors, others have distinct and cancer-subtype specific expression profiles [9–12]. Thus, it is essential to characterize the expression of more CT antigens to provide additional targets for treatment of different types of human cancer. To this end, we have identified antibodies suitable for immunostaining of the three novel CT antigens ADAM2, CALR3 and SAGE1, and characterized the expression of these proteins in normal tissues and the two most common types of human malignancies, breast and lung cancer.

## Materials and Methods

### Tissue specimens

Samples of normal tissues (skin, tonsil, esophagus, salivary gland, lung, thyroid, spleen, thymus, liver, gall bladder, kidney, pancreas, cerebellum, uterus, placenta, muscle, testis, prostate, bladder, colon, duodenum, ventricle) were collected as diagnostic specimens from patients treated at the University Hospital of Odense. The ethical committee of Funen and Vejle County (VF20050069) approved the use of these tissues, without informed consent from participants. The lung (LC1502) and breast (BRC1502) carcinoma tissue microarrays were purchased from BioCat GmbH, Heidelberg, Germany. The lung carcinoma tissue microarray LC1502 contained 23 cases of lung squamous cell carcinoma, 21 lung adenocarcinoma, 5 each of lung adenosquamous carcinoma and bronchioalveolar carcinoma, 7 small cell undifferentiated lung carcinoma, 1 each undifferentiated lung carcinoma and malignant mesothelioma, 2 each of large cell lung carcinoma and carcinosarcoma, 3 neuroendocrine lung carcinoma, and 1 each of lung chronic bronchitis, lobar pneumonia and pulmonary tuberculosis, 2 normal lung tissue, duplicate cores per case (duplicated cores from the same patient were put onto upper and lower rows in the same position). The breast carcinoma tissue microarray BRC1502 contained 62 cases of ductal carcinoma, 2 lobular carcinoma and 1 each of papillary carcinoma, sarcoma, mucinous adenocarcinoma and tubular carcinoma. The estrogen receptor and HER2 status of this first cohort of breast cancers were not available. Tissue microarrays of the second cohort of breast cancers with information on receptor status were subsequently analyzed. The generation and characterization of this second cohort has previously been reported [13]. The experiment was conducted in compliance with the Helsinki declaration.

### Immunohistochemical staining

Paraffin-embedded, formalin-fixed tissues were cut in 6 μm sections, deparaffinized, treated with 1.5% H_2_0_2_ in Tris-buffered saline (pH 7.5) for 10 min to block endogenous peroxidase activity. Thereafter, they were rinsed in distilled H_2_O, demasked, processed for antigen retrieval and washed in TNT buffer (0.1 M Tris, 0.15 M NaCl, 0.05% Tween-20, pH 7.5). To optimize conditions for staining with antibodies raised against amino acids 122–215 of ADAM2 (rabbit polyclonal, HPA026581; Sigma Aldrich, Brøndby), amino acids 195–294 of CALR3 (rabbit polyclonal, NBP2-33524, Novus, Littleton, CO, USA) and amino acids 497–641 of SAGE1 (rabbit polyclonal, HPA003208, Sigma Aldrich), different concentrations were tested in combination with different antigen retrieval protocols using sections of human testis. The methods of antigen retrieval included microwave boiling for 15 min in 1) T-EG buffer (10mM Tris, 0.5 mM EGTA, pH 9.0), 2) 10 mM citate buffer, pH 6.0 or 3) Dako Target retrieval solution (Dako S1699), or subjected to proteolytic treatment using 4) 0.05% protease type XIV (pronase E, Sigma, cat. no. P5147) in TBS, pH 7.0 for 15 min at 37°C, or 5) 0.4% pepsin (Sigma, cat. no. P7012) in 0.01M HCl for 20 min at 37°C. A protocol for immunohistochemical staining with mouse anti-MAGE-A (mouse monoclonal, Clone 6C1 Santa Cruz Biotechnology, Heidelberg, Germany; recognizing MAGE-A1, -A2, -A3, -A4, -A6, -A10 and –A12) has previously been described [9]. Primary antibodies were diluted in antibody diluent (S2022, DAKO Cytomation, Glostrup, Denmark) for 1h at room temperature. Sections were washed with TNT and incubated with horseradish peroxidase-conjugated “Ready-to-use” EnVision+ polymer K4001 (DAKO Cytomation) for 30 min, followed by another wash with TNT. The final reaction product was visualized by incubating with 3,3’-diaminobenzidine (DAB)+ substrate-chromogen for 10 min, followed by washing with H_2_O and counterstaining of sections with Mayers hematoxylin before mounting in AquaTex (Merck Inc., Whitehouse Station, NJ, USA).

### Histological evaluation

Tissue sections were evaluated by immunohistochemistry and considered positive if staining was observed in more than 5 cells, regardless of intensity.

### Cell culture

Breast (MDA-MB-231, MCF-7, T47D) and lung cancer cell lines (HCC827, PC9, A549) were cultured in RPMI or DMEM (Life technologies, Naerum, Denmark) supplemented with 10% fetal bovine serum (Sigma Aldrich), penicillin (100 U/ml) and streptomycin (100 mg/ml). For MCF7 and T47D, 6–8 ng/ml insulin (Sigma Aldrich) was added to the media. When indicated, cells were grown with 5μM 5-Aza-2’-deoxycytidine (Sigma-Aldrich) for 48 hours. Cell lines were obtained from ATCC, Wesel, Germany.

### Quantitative PCR

Total RNA was purified from cultured cells using Isol-RNA lysis reagent (5 Prime, Hilden, Germany) and RevertAid Premium Reverse Transcriptase kit (Fermentas/Life Technologies) was used for cDNA synthesis with random hexamer primers. Relative quantification of gene expression was performed with SYBR green PCR Mastermix (Applied Biosystems, Nearum, Denmark). PCR primers were: ADAM2 (Qiagen, Copenhagen, Denmark, QT00039130), CALR3 (Qiagen, QT00036162), SAGE1 (Qiagen, QT00044422) and PUM1 (Qiagen, QT00029421).

## Results and Discussion

**Fig 1:**
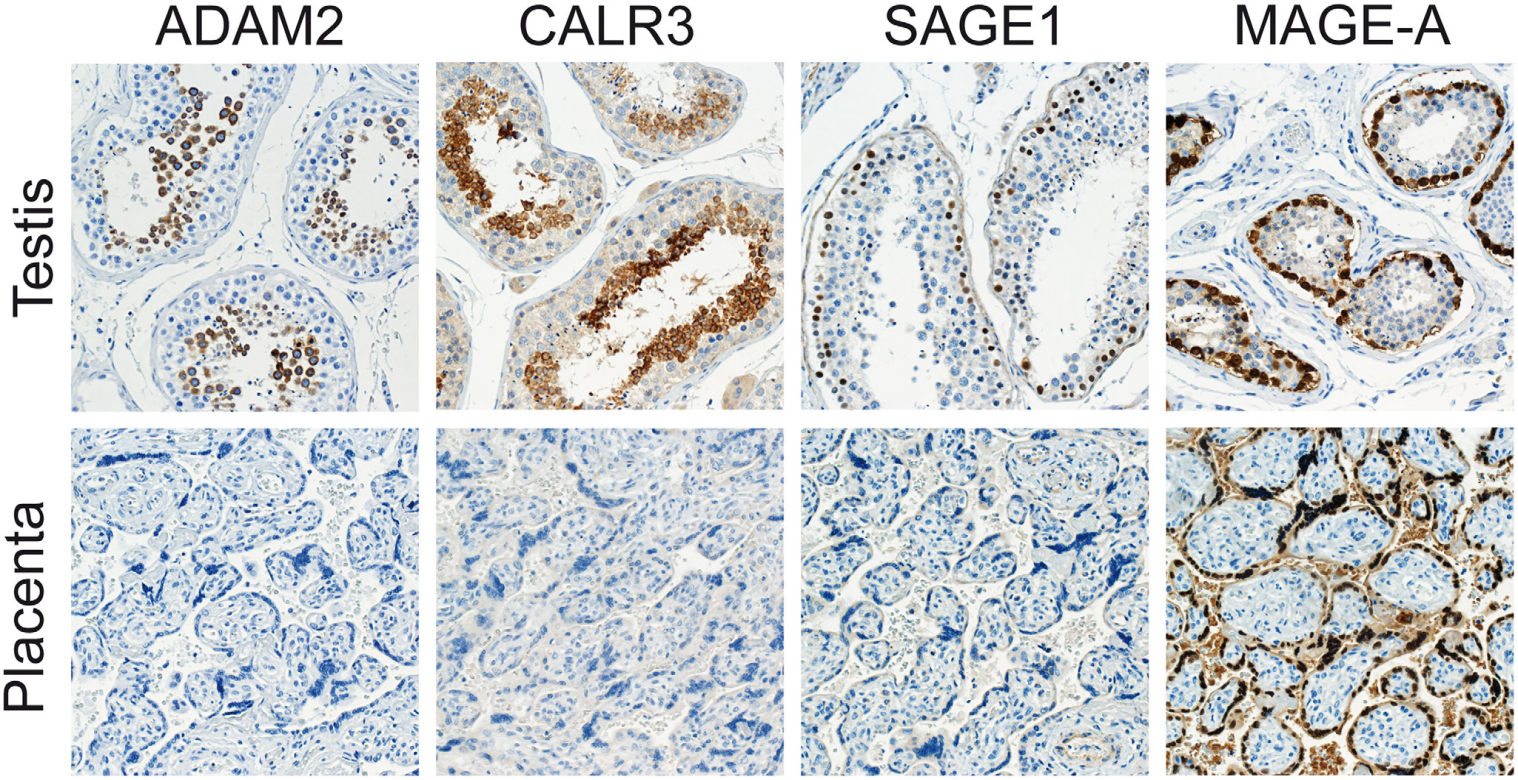
Immunohistochemical staining of CT antigens in normal tissues. Among 22 normal tissues (see materials and methods), ADAM2, CALR3, SAGE1 and MAGE-A proteins were only detected in the germ cells of the testis. More specifically, SAGE-1 and MAGE-A were found in spermatogonia, while ADAM2 and CALR3 were present in post-meiotic germ cells. Only MAGE-A proteins were detected in plancenta (all pictures magnification x20).

Lung and breast cancer are the two most common types of cancer, and novel therapeutic strategies are needed to optimize clinical treatments. To identify potential targets for immunotherapy of lung and breast cancer, we used a panel of previously uncharacterized antibodies to investigate the expression of three previously uncharacterized CT antigens, ADAM2, CALR3 and SAGE1, in patient tumors, cancer cell lines and a panel of normal tissues. The antibodies used were expected to detect all variants of the target proteins. For comparison, we included a well-characterized antibody recognizing MAGE-A CT antigens. Conditions for immunohistochemical analysis of ADAM2, CALR3 and SAGE1 were optimized with regard to method of antigen retrieval and antibody concentration using tissue sections of normal testis (see method for details). Further investigation of ADAM2, CALR3 and SAGE1 in 22 normal tissues demonstrated that these proteins were selectively expressed in testis germ cells (Fig 1). ADAM2 and CALR3 were expressed at the spermatocyte and spermatid stage of spermatogenesis and were both localized at the cell membranes. SAGE1, on the other hand, was limited to the spermatogonia and pre-meiotic spermatocytes and were localized to the nuclei. These results are consistent with previous reports suggesting that chromosome X-encoded CT antigens, such as SAGE-1, are generally expressed in pre-meiotic stages of germ cells, while autosomal-encoded CT antigens, such as ADAM2 and CALR3, are generally expressed in post-meiotic stages of germ cells [12]. No expression of ADAM2, CALR3 and SAGE1 was seen in placenta (consistent with available RT-PCR results from the CTDatabase, http://www.cta.lncc.br), in contrast to MAGE-A (Fig 1) and other CT antigens [12]. These results suggested that ADAM2, CALR3 and SAGE1 were testis-specific in expression and supported previous analyses of mRNA levels in normal tissues [14, 15], further highlighting their potential as specific cancer targets. The importance of targeting antigens with high cancer-specificity has recently been emphasized by two studies with genetically modified T-cell receptors wherein patients died due to unexpected reactivity towards non-cancerous tissues [16–18].

**Fig 2:**
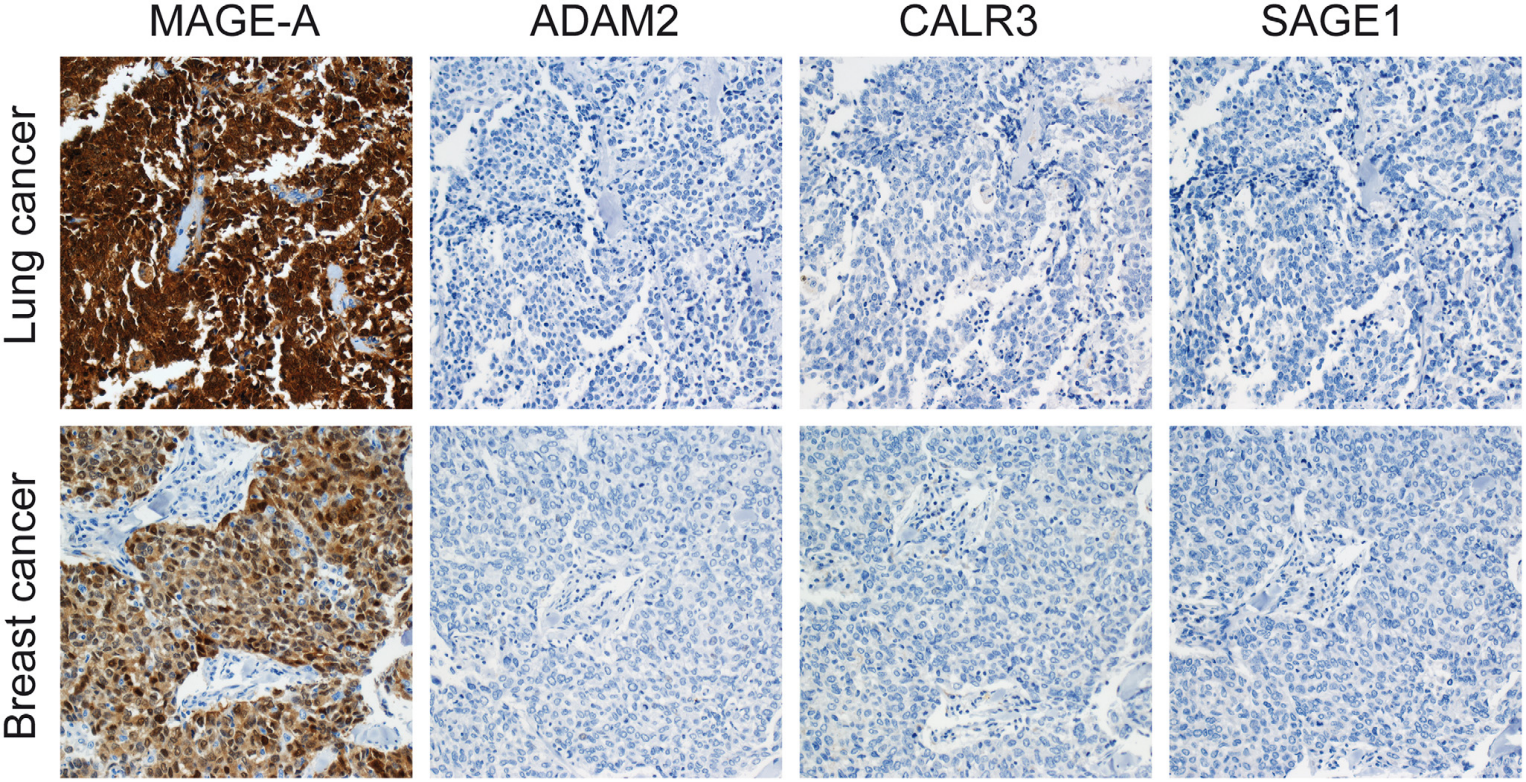
Immunohistochemical staining of CT antigens in lung and breast cancer tumors. Examples of lung and breast cancer tumors positive for MAGE-A and negative for ADAM2, CALR3 and SAGE1 (all pictures magnification x20).

Having established the ability of the antibody reagents to specifically detect ADAM2, CALR3 and SAGE1 CT antigens, we next investigated the expression of these proteins in primary tumors from patients with different subtypes of lung cancer at different stages. The panel included 55 non-small cell lung cancers (NSCLC, including squamous cell carcinomas, adenocarcinomas, adenosquamous carcinoma, bronchioalveolar carcinoma, undifferentiated lung carcinoma, mesothelioma, neuroendocrine lung carcinoma and large cell carcinomas), which constitute about 85% of lung cancers (Table 1; Fig 2). A number of small cell lung cancers as well as some more rare lung cancer subtypes were also included in the analysis, however, the relatively low numbers of samples from these subtype did not allow statistically sound conclusions. Surprisingly, we found that none of these tumors expressed any of the three CT antigens. In contrast, 16% (confidence interval; +/-8.8) of the lung cancers (11/67) were positive for MAGE-A CT antigens, in agreement with earlier studies [9, 19]. Other CT antigens are also expressed at relatively high frequencies in lung cancer [20, 21]. This is the first report on ADAM2, CALR3 and SAGE1 expression in lung cancer.

**Table 1:** ADAM2, CALR3, SAGE1 and MAGE-A expression in lung cancer.

Stage	Number of tumors	ADAM2-positive	CALR3-positive	SAGE1-positive	MAGE-A-positive
**I**	45	0	0	0	5 (11%)
**II**	17	0	0	0	4 (24%)
**IIIa+b**	5	0	0	0	2 (40%)
**Total**	67	0	0	0	11 (16%)

**Fig 3:**
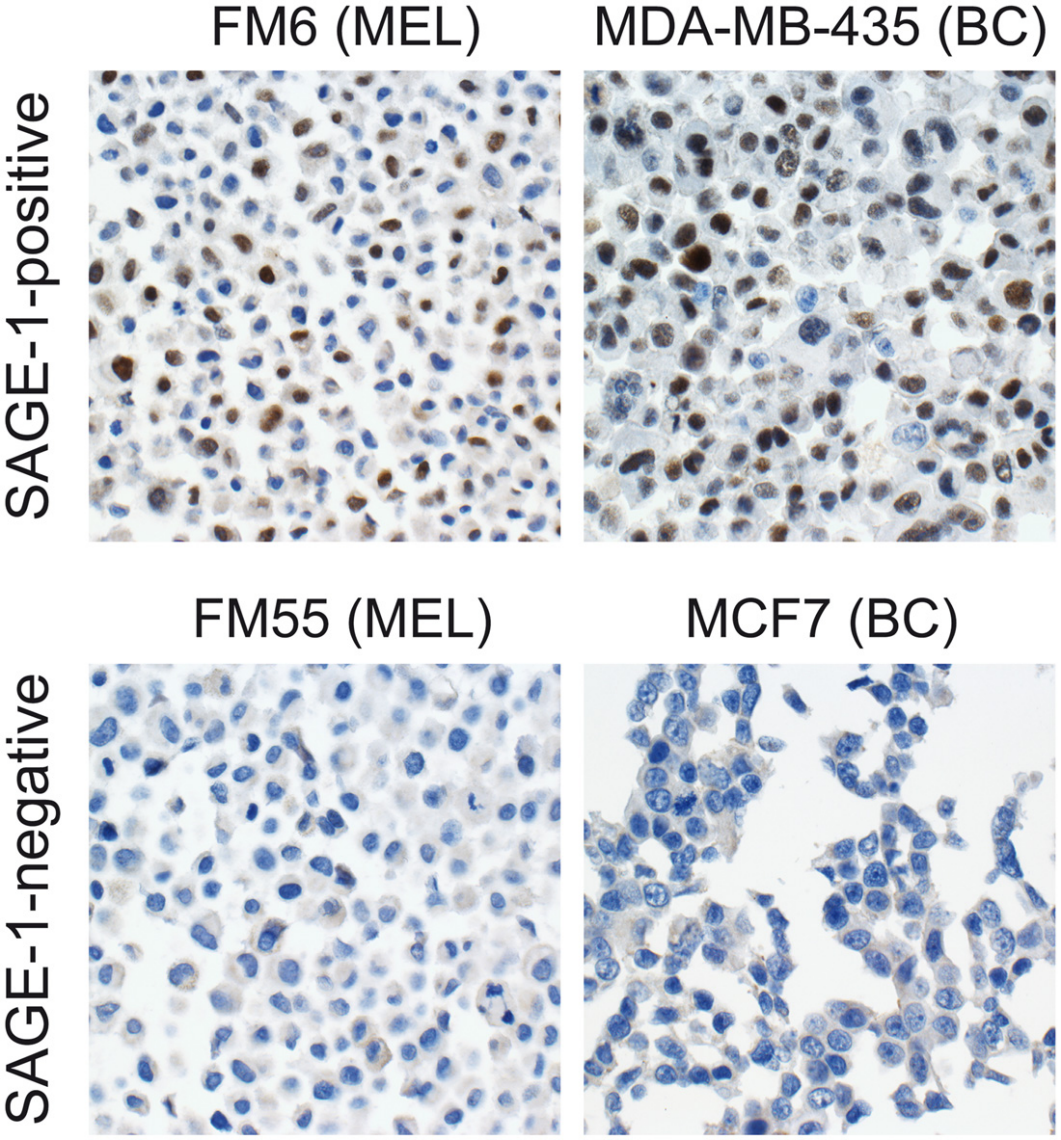
Immunohistochemical staining of SAGE1 in breast cancer and melanoma cell lines. SAGE1 was detected in the nucleus of FM6 and MDA-MB-435, while other cells lines were negative (all pictures magnification x20).

We also investigated the expression of ADAM2, CALR3 and SAGE1 in 13 breast cancer cell lines (S1 Table) and in breast cancer tumors from two cohorts of patients with data on clinical stage and receptor status, respectively (Tables 2 and 3). Nuclear staining for SAGE1 was observed in the breast cancer cell line (MB-MDA-435, debated also to be of melanoma origin, although the overwhelming studies support it being of breast cancer origin [22]) (Fig 3), while ADAM2, CALR3 was not detected in this cell line. The remaining breast cancer cell lines and the 189 breast tumors did not express the three CT antigens. MAGE-A CT antigens were expressed in 4/13 cell lines and 7% (+/- 6.1) of primary tumors of different subtypes, comparable to previous studies [23]. MAGE-A CT antigens were more frequently expressed in estrogen receptor-negative tumors (18% +/- 6.9) consistent with other reports [24, 25].

**Table 2:** Analysis of ADAM2, CALR3, SAGE1 and MAGE-A expression in cohort 1 consistent of 68 breast cancers of different clinical stages.

Stage	Number of tumors	ADAM2-positive	CALR3-positive	SAGE1-positive	MAGE-A-positive
**I**	8	0	0	0	0
**I-II**	5	0	0	0	0
**II**	26	0	0	0	3 (12%)
**II-III**	24	0	0	0	2 (8%)
**III**	5	0	0	0	0
**Total**	68	0	0	0	5 (7%)

**Table 3:** Analysis of ADAM2, CALR3 and MAGE-A expression in cohort 2 consisting of 121 breast cancers of different receptor status.

Receptor status	Number of tumors	ADAM-positive	CALR3-positive	SAGE1-positive	MAGE-A-positive
ER-positive	18	0	0	ND	1 (6%)
ER-negative	103	0	0	ND	18 (18%)
HER2-positive	40	0	0	ND	7 (18%)
HER-negative	81	0	0	ND	12 (15%)
ER- and HER2-negative	70	0	0	ND	11 (16%)
All	121	0	0	ND	19 (16%)

ND = not determined (staining of these tissue arrays with the SAGE1 antibody produced significant background coloration resulting in inconclusive results).

The absence of ADAM2, CALR3 and SAGE1 expression in lung and breast cancer prompted us to test their expression in a panel of melanoma cell lines (S2 Table; Fig 3), since most characterized CT antigens have shown the highest incidence in melanoma among the different types of cancer. SAGE-1 was expressed in 6/17 melanoma cell lines, while ADAM2 and CALR3 were not detected in any of the 17 cell lines (S2 Table).

**Fig 4:**
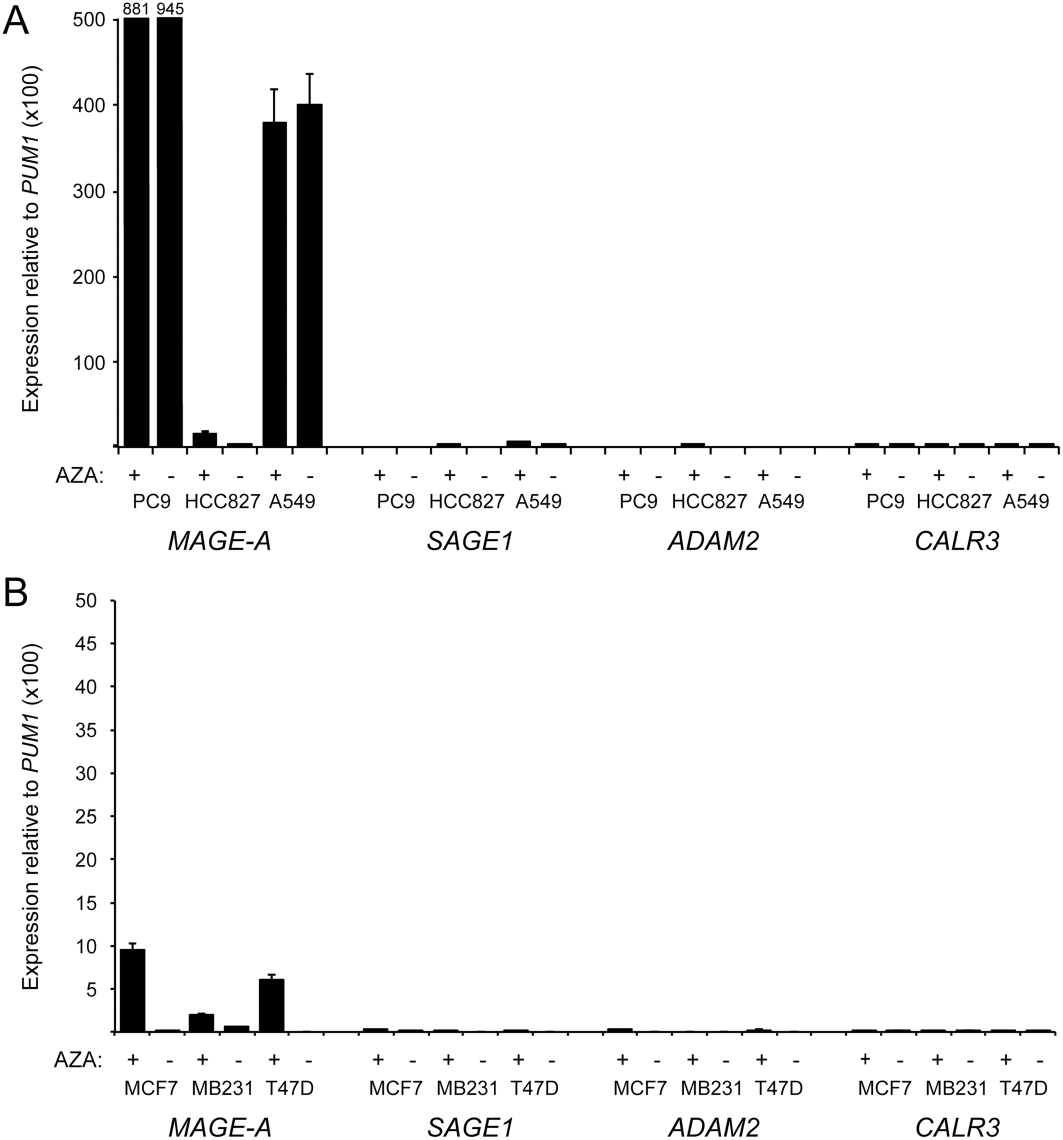
Effect of 5-aza-2’-deoxycytidine-treatent on the expression of CT antigens in lung and breast cancer cell lines. Expression of MAGE-A1, ADAM2, CALR3 and SAGE1 CT antigen genes in 5-aza-2’-deoxycytidine-treated (5 μM, 48 hours) and untreated lung (A) and breast (B) cancer cell lines was measured using quantitative PCR. Error bars = standard deviation.

Infrequent or heterogeneous expression of tumor antigens in tumors may be an obstacle to development of efficient and widely applicable cancer vaccines. Importantly, agents that inhibit the function of DNA methyltransferases have been demonstrated to induce the expression of CT antigen genes. For instance, 5-aza-2’-deoxycytidine activates MAGE-A, GAGE, CT45 and many other CT antigen genes [3, 26–29]. To investigate the possibility of inducing ADAM2, CALR3 and SAGE1 expression in lung and breast cancer cells, we stimulated lung (PC9, HCC827 and A549) and breast cancer cell lines (MCF7, MDA-MB-231 and T47D) with 5-aza-2’-deoxycytidine (Fig 4). Levels of MAGE-A gene expression increased significantly in all cell lines, except for PC9 and A549, which already exhibited high MAGE-A expression. In contrast, neither ADAM2, CALR3 nor SAGE1 expression was significantly increased in any of the cell lines, suggesting that DNMT inhibition cannot potentiate immunotherapy targeting these antigens.

## Conclusions

We investigated the potential of three previously uncharacterized CT antigens, ADAM2, CALR3 and SAGE1, as targets for treatment of lung and breast cancer. Although we demonstrated that these CT antigens exhibit a germ cell-specific expression pattern in normal tissues, which is an important feature of antigens for cancer immunotherapy, none of these antigens were expressed in lung and breast cancer. Furthermore, the expression of these antigens could not be induced by DNMT-inhibitor treatment, in contrast to many other CT antigens. In conclusion, ADAM2, CALR3 and SAGE1 should not be considered targets for treatment of lung and breast cancer. However, our study also provides the reagents and conditions for assessing expression of these antigens in other cancer types. Furthermore, SAGE1 was detected in about 40% of melanoma cell lines, suggesting that this protein may be an interesting target. Our study represents an important step in the tedious evaluation of the long list of potential tumor antigens to identify novel therapeutic targets for different types of cancer.

## Supporting Information

S1 Table ADAM2, CALR3, SAGE1 and MAGE-A expression in breast cancer cell lines. (DOCX)

S2 Table ADAM2, CALR3, SAGE1 and MAGE-A expression in melanoma cells lines. (DOCX)
