# Supplementary material for: Lack of ADAM2, CALR3 and SAGE1 Cancer/Testis Antigen Expression in Lung and Breast Cancer
Source: PLoS One. 2015 Aug 7;10(8):e0134967. doi: 10.1371/journal.pone.0134967 (PMC4529184; doi:10.1371/journal.pone.0134967)
Supplement: S1 Table — (DOCX) [file pone.0134967.s001.docx]

**Table S1. ADAM2, CALR3, SAGE1 and MAGE-A expression in breast cancer cell lines**

| **Cell line** | **ADAM2-**  **positive** | **CALR3-**  **positive** | **SAGE1-**  **positive** | **MAGE-A-**  **positive** |
| --- | --- | --- | --- | --- |
| **BT20** | - | - | - | - |
| **MDA-MB-157** | - | - | - | - |
| **MDA-MB-231** | - | - | - | - |
| **MDA-MB-435** | - | - | +++ | +++ |
| **MDA-MB-468** | - | - | - | - |
| **T47D** | - | - | - | ++ |
| **Hs578T** | - | - | - | ++ |
| **MCF7** | - | - | - | - |
| **ZR-75-1** | - | - | - | - |
| **BT474** | - | - | - | - |
| **SK-BR-3** | - | - | - | ++ |
| **CAL-51** | - | - | - | - |
| **BrCa-MZ-01** | - | - | - | - |
