# Supplementary material for: Lack of ADAM2, CALR3 and SAGE1 Cancer/Testis Antigen Expression in Lung and Breast Cancer
Source: PLoS One. 2015 Aug 7;10(8):e0134967. doi: 10.1371/journal.pone.0134967 (PMC4529184; doi:10.1371/journal.pone.0134967)
Supplement: S2 Table — (DOCX) [file pone.0134967.s002.docx]

**Table S2. ADAM2, CALR3, SAGE1 and MAGE-A expression in melanoma cells lines**

| **Cell line** | **ADAM2-**  **positive** | **CALR3-**  **positive** | **SAGE1-**  **positive** | **MAGE-A-**  **positive** |
| --- | --- | --- | --- | --- |
| **SK-MEL-28** | - | - | + | +++ |
| **SK-MEL-37B** | - | - | - | +++ |
| **SK-MEL-44** | - | - | +++ | +++ |
| **MZ2** | - | - | - | +++ |
| **FM2** | - | - | - | +++ |
| **FM3** | - | - | + | + |
| **FM6** | - | - | ++ | +++ |
| **FM28** | - | - | +++ | +++ |
| **FM45** | - | - | ++ | +++ |
| **FM55** | - | - | - | + |
| **FM57** | - | - | - | - |
| **FM72** | - | - | - | +++ |
| **FM79** | - | - | - | +++ |
| **FM81** | - | - | - | ++ |
| **FM82** | - | - | - | +++ |
| **FM86** | - | - | ++ | + |
| **FM88** | - | - | - | - |
